# High-output cardiac failure due to multiple femoral arteriovenous fistulae

**DOI:** 10.11604/pamj.2020.37.246.25099

**Published:** 2020-11-18

**Authors:** Mogamat-Yazied Chothia, Amaan Panday

**Affiliations:** 1 Division of General Medicine, Department of Medicine, Faculty of Medicine and Health Sciences, Stellenbosch University and Tygerberg Hospital, Cape Town, South Africa

**Keywords:** Tuberculous, arteritis, HIV

## Image in medicine

A 39-year-old woman presented with symptoms of dyspnea, orthopnea, paroxysmal nocturnal dyspnea, weight loss, night sweats and fatigue that were present for 3-months. She had been diagnosed with human immunodeficiency virus (HIV) infection in 2016 but had defaulted her antiretroviral therapy. She had a low CD4 count of 17 cells/μl and her HIV viral load was log 6.16 copies/ml. She had evidence of high-output cardiac failure on clinical examination that included a wide pulse pressure with a blood pressure of 138/59 mmHg, radial pulse had a collapsing character and the pulse rate was 125 beats per minute, jugular venous distention was observed, the apical impulse was displaced with no audible murmurs and bibasilar crackles were present. She had a thrill palpable over the right femoral artery and a continuous bruit was audible. Significant laboratory results included a hemoglobin concentration of 6.7 g/dL and an erythrocyte sedimentation rate of more than 140 mm/hour. Importantly, rapid plasma reagin, anti-nuclear antibodies and anti-neutrophil cytoplasmic antibodies were negative. Computed tomography angiogram (CT angiogram) revealed fusiform aneurysmal dilatation of the distal abdominal aorta and right common iliac artery with multiple right lower limb arteriovenous fistulae and venous aneurysms. There were no other clinical or radiological findings to suggest a large-vessel vasculitis such as Takayasu´s arteritis. *Mycobacterium tuberculosis* was cultured from multiple sources including a gastric aspirate, blood and sputum samples. A diagnosis of tuberculous arteritis was made. Anti-tuberculous and heart failure treatment were commenced and her antiretroviral therapy was reinitiated. She reported significant improvement of her symptoms. However, 1-week following hospital discharge, the patient demised.

**Figure 1:**
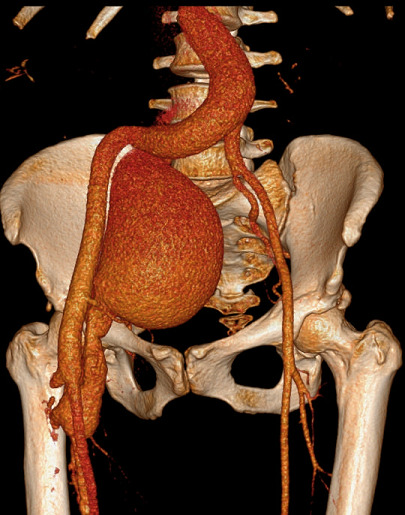
computed tomography angiogram

